# Decoy Technology as a Promising Therapeutic Tool for Atherosclerosis

**DOI:** 10.3390/ijms22094420

**Published:** 2021-04-23

**Authors:** Maryam Mahjoubin-Tehran, Yong Teng, Amin Jalili, Seyed Hamid Aghaee-Bakhtiari, Alexander M. Markin, Amirhossein Sahebkar

**Affiliations:** 1 Department of Medical Biotechnology and Nanotechnology, Faculty of Medicine, Mashhad University of Medical Sciences, Mashhad 9177948564, Iran; mahjoubintm951@mums.ac.ir (M.M.-T.); jalilia@mums.ac.ir (A.J.); 2 Department of Oral Biology and Diagnostic Sciences, Dental College of Georgia, Augusta University, Augusta, GA 30912, USA; yteng@augusta.edu; 3 Bioinformatics Research Group, Mashhad University of Medical Sciences, Mashhad 9177948564, Iran; aghaeibh@mums.ac.ir; 4 Laboratory of Cellular and Molecular Pathology of Cardiovascular System, Institute of Human Morphology, 3 Tsyurupa Street, 117418 Moscow, Russia; alexander.markin.34@gmail.com; 5 Applied Biomedical Research Center, Mashhad University of Medical Sciences, Mashhad 9177948564, Iran; 6 Biotechnology Research Center, Pharmaceutical Technology Institute, Mashhad University of Medical Sciences, Mashhad 9177948564, Iran; 7 School of Pharmacy, Mashhad University of Medical Sciences, Mashhad 9177948564, Iran

**Keywords:** decoy, atherosclerosis, cardiovascular disease

## Abstract

Cardiovascular diseases (CVDs) have been classified into several types of disease, of which atherosclerosis is the most prevalent. Atherosclerosis is characterized as an inflammatory chronic disease which is caused by the formation of lesions in the arterial wall. Subsequently, lesion progression and disruption ultimately lead to heart disease and stroke. The development of atherosclerosis is the underlying cause of approximately 50% of all deaths in westernized societies. Countless studies have aimed to improve therapeutic approaches for atherosclerosis treatment; however, it remains high on the global list of challenges toward healthy and long lives. Some patients with familial hypercholesterolemia could not get intended LDL-C goals even with high doses of traditional therapies such as statins, with many of them being unable to tolerate statins because of the harsh side effects. Furthermore, even in patients achieving target LDL-C levels, the residual risk of traditional therapies is still significant thus highlighting the necessity of ongoing research for more effective therapeutic approaches with minimal side effects. Decoy-based drug candidates represent an opportunity to inhibit regulatory pathways that promote atherosclerosis. In this review, the potential roles of decoys in the treatment of atherosclerosis were described based on the in vitro and in vivo findings.

## 1. Introduction

Atherosclerosis is a leading etiology of cardiovascular disease (CVD). Atherosclerosis is characterized as an inflammatory process in the arterial wall that can lead to artery diseases and stroke [1,2]. Atherosclerosis underlies several important vascular pathological condition events such as peripheral arterial diseases and coronary diseases, which cause most morbidity and mortality from CVD. Cholesterol is a critical component of atherosclerotic plaques, which promotes foam cell formation and perpetuates atherosclerosis development. High plasma levels of pro-atherogenic lipoproteins such as low-density lipoprotein (LDL) and very low-density lipoprotein (VLDL) as well as VLDL remnants are considered among primary risk factors for the progression of atherosclerosis [3,4]. Atherosclerotic CVD mainly targets the vessels in the heart and brain resulting in ischemic heart disease and ischemic stroke [5,6]. Ischemic heart disease and stroke are presented as the first and fifth causes of death globally, respectively [7,8]. Many clinical and experimental studies have been performed to develop drugs for atherosclerosis.

## 2. Therapeutic Approaches for Atherosclerosis

### 2.1. Lipid-Lowering Therapies

Lipid-lowering therapy is the major current strategy in managing atherosclerotic cardiovascular disease. LDL is an important atherosclerotic risk factor lying at the root of human atherosclerosis. Many attempts to develop drugs for atherosclerosis have been designed to reduce the plasma level of LDL cholesterol, as well as other detrimental lipids and lipoproteins including triglyceride-rich lipoproteins [9]. Statins are the cornerstone of lipid-lowering therapy owing to their LDL-lowering and pleiotropic effects [10,11,12,13]. However, these drugs are not without adverse effects. For instance, muscle symptoms in extreme cases can lead to complete drug intolerance and discontinuation [14].

### 2.2. Antioxidant Interventions

Almost all schemes of the pathogenesis of atherosclerosis invoke oxidative modified LDL as a key instigating factor. Various phospholipases can liberate lipid moieties from oxidized lipoproteins that can activate deleterious functions of vascular cells and leukocytes found in atheromata. Yet, several attempts to inhibit phospholipases to prevent the generation of these putatively pernicious mediators have not borne fruit [15].

### 2.3. Anti-Inflammatory Interventions

The role of inflammatory pathways in the pathogenesis of atherosclerosis has been demonstrated. Some studies were designed to target various inflammatory pathways in secondary prevention of atherosclerotic events [16]. A hallmark of inflammation is the recruitment of leukocytes, which is a process depending on endothelial-leukocyte expression. In addition to endothelial cells, platelets contain one such adhesion molecule, P-selectin. This double source points to a pivotal role of this particular adhesion molecule in acute inflammation complicated by thrombosis [17]. Moreover, the (NOD-, LRR-, and pyrin domain-containing protein 3) (NLRP3) inflammasome regulates the activity of its constituent protein caspase-1. The inflammasome undergoes activation by a number of pathogen-associated molecular patterns and damage-associated molecular patterns. Several atherosclerosis-related stimuli also can coactivate the NLRP3 inflammasome [15].

### 2.4. Potential Vaccinations

Scientific evidence during the past 40 years has implicated an adaptive immune response against plaque-associated autoantigens in atherogenesis. Preclinical data have underscored the protective potential of immunization against such targets precisely [18]. Existence of both proatherogenic and athero-protective immune responses have led to the notion that suppressing proatherogenic immune response or activation of an athero-protective immune response may be beneficial for atherosclerosis. These pathophysiological principles hence raise the tantalizing possibility that immune modulation using a vaccine or specific antibody could favorably alter the natural history of atherosclerosis. Modifications of the immune response using a vaccine containing antigen(s) relevant to atherogenesis may promote specific immune responses against relevant antigens with the potential for a more sustained effect, but not affect the global immunity [19].

Despite the efficacy of conventional drugs such as statins, there is limited response in certain groups such as patients with familial hypercholesterolemia patients [20]. In addition, many statin-treated patients remain at a high risk of residual CVD risk despite reaching LDL-cholesterol goals [3]. Furthermore, these drugs have many side effects such as elevation of liver enzyme concentrations [21], drug–drug interactions [22], and myopathy disorders [23]. Thereby, the search for more effective drugs with fewer side effects for atherosclerosis treatment has continued.

In addition to protein-based therapeutics (recombinant proteins and antibodies), efforts have been made toward the development of nucleic acid-based drugs which are stable and have longer half-lives than protein-based therapeutics [24,25]. The nucleic acid-based therapeutic approach includes antisense oligonucleotides (ASO), small interfering RNAs (siRNA), MicroRNAs (miRNA), and decoys [26].

## 3. Decoy

### 3.1. Decoy Oligodeoxynucleotides (ODN)

**Figure 1:**
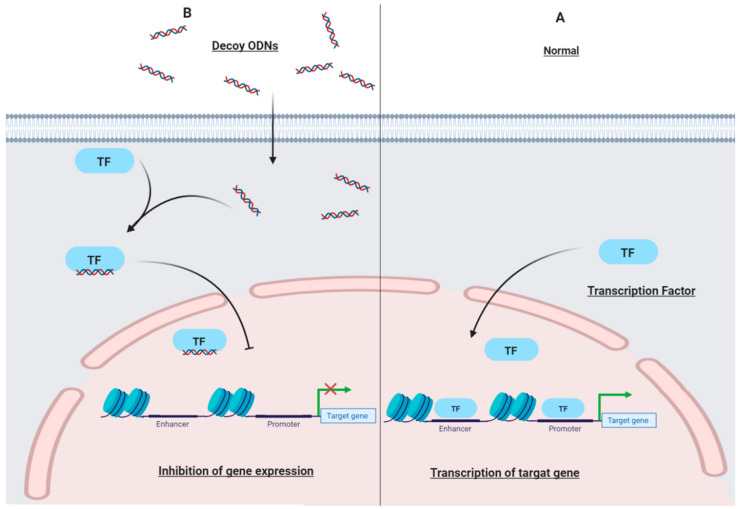
The mechanism of action of decoy ODNs. (**A**) Without decoy ODNs: normal transcription of target genes. (**B**) In the presence of decoy ODNs: decoy ODNs bind to a specific transcription factor. Despite entering the nucleus, the transcription factor could not bind to the specific region and promote transcription of the target gene. ODNs: oligodeoxynucleotides; TF: transcription factor.

Decoy ODNs are short double-stranded deoxyribonucleic acid (DNA) that have a similar sequence as DNA-binding site of transcription factors (TF) and can selectively block TF activation of target genes [27]. Decoy ODN technology aims to inhibit expression of target genes at the transcriptional level through competition for trans elements of the regulatory regions of genes (Figure 1) [28,29,30]. Due to the specificity, finding the best sequence of TF binding sites for regulation of the target gene expression is one of the technical obstacles of this method [31]. The use of decoy ODNs as a therapeutic tool has been established for many years for various diseases including cancers [32]. Decoy transfection is a promising tool for inhibition of gene activation. Although the decoy could be synthesized easily, the effectiveness of in vivo delivery remains difficult [33]. Moreover, a great deal of research has been focused on improving pharmacological characteristics such as biostability, resistance to serum nucleases, cell adsorption, and nuclear localization of decoy ODNs by modifications such as peptide nucleic acids, chimeric decoy oligonucleotides, and locked nucleic acids [29].

### 3.2. Decoy Peptide

**Figure 2:**
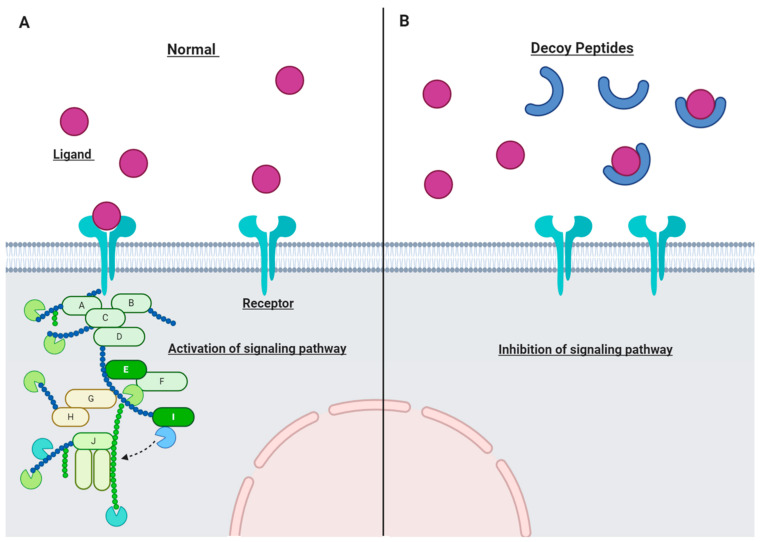
The mechanism of action of decoy peptides. (**A**) Normal cell without decoy peptides. (**B**) Decoy peptides act as molecular traps and bind to the specific ligands; so, the corresponding signaling pathway would be inhibited.

Decoy peptides (or decoy receptors) are "sticky" proteins similar to functional receptors on cytokines and chemokines [34]. Decoy peptides are specifically designed to attract certain ligands such as inflammatory cytokines and compete with target receptors although decoy peptides do not initiate signaling (Figure 2) [35]. The decoy peptide is designed to trap the specific ligand molecules as efficiently as their respective receptor. Therefore, ligand binding to the functional receptor is inhibited and the corresponding signaling pathway is suppressed.

Decoy peptides are applied throughout various fields of research and have been demonstrated as an anticancer agent via a decoy receptor for Wnt ligands [34,36]. Importantly, decoy receptors constitute a novel and relatively unknown target for development of antiviral drugs [37] or as a therapeutic tool for inhibition of bacterial infections [38]. Moreover, decoy peptides are employed as a novel class of therapeutics for combatting neurotoxic envenoming [39]. Interestingly, decoy peptides bind to oligomeric and fibrillar amyloid β with high affinity, form complexes, and block amyloid β toxicity in Alzheimer’s disease [40].

## 4. Targets

### 4.1. Protein Phosphatase 1

Abnormalities in vascular smooth muscle cell (VSMC) proliferation are an important etipathological factor for vascular proliferative disorders. The proliferation of VSMC is associated with a chronic increase in the cytosolic Ca^2+^ level, which is caused by the loss of Ca^2+^ handling proteins, such as sarco/endoplasmic reticulum Ca^2+^-ATPase (SERCA2a) [41]. The gene transfer-mediated restoration of the SERCA2a level attenuates VSMC proliferation and neointimal formation [42]. Therefore, the maintenance of a low cytosolic Ca^2+^ level by controlling SERCA2a activity is a reasonable strategy to prevent VSMC proliferation. SERCA2a activity is inhibited by a direct interaction with phospholamban, whose inhibitory activity is enhanced by dephosphorylation by protein phosphatase 1 (PP1) [43]. Therefore, the inhibition of the PP1-mediated dephosphorylation of PLB is a promising approach to upregulate SERCA2a activity [44].

Jang et al. used the peptide decoy, named ψPLB-SE, to target protein phosphatase 1 in cardiomyocytes and thereby normalize the activity of SERCA2a. Their results indicated that ψPLB-SE reduced neointimal growth in balloon-injured rat carotid arteries, as well as VSMC proliferation and migration. Furthermore, ψPLB-SE could also inhibit SERCA2a degradation in injured rat carotid arteries. They concluded that ψPLB-SE could correct the handling of abnormal Ca2+ by activating SERCA2a [44].

### 4.2. Macrophage Scavenger Receptors

Macrophage scavenger receptors (MSRs) induce the formation of atherosclerotic lesions. MSRs can efficiently bind and internalize atherogenic modified LDL such as acetylated LDL and oxidized LDL in the vessel wall causing the lipids deposition in the arterial wall. Furthermore, they facilitate cation-independent adhesion of macrophages and promote lesion development [45]. Since MSRs are not downregulated by the accumulation of lipids in the cell, accumulation of lipids in the lesion macrophages continues [45]. Soluble decoy receptors have the potential to be effective tools for inhibiting receptor-mediated functions [46].

Jalkanen and colleagues generated a modified MSR known as a secreted macrophage scavenger receptor (sMSR), which contained the human MSR AI extracellular domains and signal sequence. Results showed that this sMSR decoy reduced the degradation of atherogenic modified LDL. Monocyte/macrophage adhesion on endothelial cells was decreased, and macrophage foam cell formation was inhibited in sMSR decoy treatment [45]. In another study for testing the long-term effects, an sMSR decoy was transduced to Western-type diet fed LDL receptor (LDLR) knockout mice via an adeno-associated virus (AAV). sMSR decoy protein was detected in the transduced mice plasma 6 months after the gene transfer. The sMSR decoy significantly reduced atherosclerotic lesions in the aorta by 21% compared to the control [46]. The sMSR decoy was also transduced to the hypercholesterolemic LDLR knockout mice via a recombinant adenovirus. The results revealed that while atherosclerotic lesions in the whole aortic area were reduced, the change in the aortic root was not statistically significant [47].

### 4.3. Activator Protein-1

VSMCs migration and proliferation are common characteristics of responses in vascular diseases with various factors being involved in the process [48]. Studies showed that in balloon-injured arteries, transcription factor activator protein-1 (AP-1) is upregulated in injured arterial smooth muscle cells [48]. Moreover, transforming growth factor-β1 (TGF-β1) has been implicated in the development of human restenosis after angioplasty as well as in neointimal lesions in balloon-injured arteries. TGF-β1 mRNA levels were found to be increased in restenotic lesions. It is worth noting that the TGF-β1 gene has an AP-1 consensus sequence in its promoter region [49]. One of the mechanisms of neointimal thickening suppression by AP-1 decoy is suppression of VSMC proliferation by blocking platelet-derived growth factor (PDGF)-mediated MAP kinase (MAPK) and AP-1 signaling pathways and TGF-β1 production [48].

Kume et al. transferred decoy ODNs containing the AP-1 binding site in balloon-injured rabbit carotid arteries. They used the hemagglutinating virus of Japan (HVJ) liposomes method in conjunction with human aortic SMCs in order to reduce AP-1 activity and examine its effects on neointimal thickening. Result showed that the decoy reduced AP-1 DNA-binding activity due to specific binding of the decoy to AP-1 in vivo as well as a reduction of the neointimal area. Furthermore, they showed that the AP-1 decoy decreased the cell number of SMCs. TGF-β1 could also reduce SMCs production under stimulation of platelet-derived growth factors. Transfection of VSMCs with this decoy prevented transactivation of some vital cell-cycle regulatory genes; in that way inhibiting proliferation of VSMC and remodeling the vascular wall. As a result, they suggested the AP-1 decoy as a novel therapeutic approach for restenosis [48].

Neointimal formation and intimal hyperplasia is the main cause of late vein graft failure; in which proliferation and migration of SMCs are the underlying mechanisms. AP-1 plays a role in the expression of many genes involved in cellular proliferation, cell cycle progression, extracellular matrix production, and neointimal formation after vascular injury.

Ahn and his associates transfected the AP-1 decoy as a therapeutic strategy for restenosis after angioplasty. The AP-1 decoy blocked proliferation and migration of VSMCs. The decoy also inhibited the formation of neointimal after balloon injury in the rat carotid artery [50]. In another study, this group evaluated the effects of the AP-1 decoy on intimal hyperplasia within a large animal model using mongrel dogs. The results indicated that the AP-1 decoy effectively inhibited intimal hyperplasia of an autogenous vein graft [51].

Xie and his coworkers investigated the effect of cardiac fibroblasts (CFs) transfection with the AP-1 decoy to determine if it would prevent proliferation of CF and expression of the matrix metalloproteinase (MMP) gene. Rat heart CFs were cultured and exposed to xanthine 1 xanthine oxidase (XXO) as well as AP-1 decoy ODNs. It was found that the decoy could inhibit XXO-induced proliferation of CF and expression of MMP genes in vitro [52].

### 4.4. Cyclic Adenosine Monophosphate Response Element

Intimal hyperplasia is a major reason for treatment failure following vascular and endovascular surgery (VES). It has been demonstrated that blunting the activation of cyclic adenosine monophosphate response element (CRE) binding protein is critical in preventing vein graft intimal hyperplasia [53].

Uchida et al. designed a CRE decoy for binding to a CRE sequence for preventing intimal hyperplasia. The results showed that the CRE decoy decreased VSMC proliferation and migration. Furthermore, the CRE decoy not only decreased CRE activity but also suppressed the intimal hyperplasia formation at injured vessel walls in mice [54].

### 4.5. Early Growth Response Factor-1

Early growth response factor-1 (Egr-1) is an important transcription factor in promoting atherosclerosis. Since Egr-1 can be quickly activated after vascular injury, it may have a critical role in vascular proliferation and inflammation [55]. The excess VSMC proliferation and the intimal hyperplasia development is a hallmark of vein graft failure. Egr-1 could contribute to vascular proliferation and inflammation. Wang et al. transfected Egr-1 decoy ODNs to the vein graft of rabbits to evaluate their effects on the thickness of the intima. They found that Egr-1 decoy ODNs decreased the expression of Egr-1 as well as reduced VSMC proliferation and intimal hyperplasia [56].

Egr-1 has a master regulatory role in cardiovascular diseases such as atherosclerosis and restenosis. Han and colleagues designed an Egr-1 decoy in balloon-injured arteria of rats. The results showed that Egr-1 decoy could not down regulate the expression of Egr-1. The Egr-1 decoy, however, proved to effectively block downstream genes such as proliferating-cell nuclear antigen (PCNA), cyclinD1, and cyclin-dependent kinases 4 (cdk4), thus inhibiting VSMC proliferation and neointimal hyperplasia [57].

An experiment conducted by Ohtani et al. evaluated the effect of the Egr-1 decoy for the treatment of atherosclerosis and restenosis. The Egr-1 decoy was quickly intraluminally transfected to the hypercholesterolemic rabbits subsequent to the carotid artery balloon injury. The results demonstrated that Egr-1 activity was increased after balloon injury but was prevented by the Egr-1 decoy. Furthermore, the Egr-1 decoy reduced the expression of Egr-1-dependent genes, such as platelet-derived growth factor-B (PDGF-B) and TGF-β1, which in turn reduced early inflammation, proliferation, and later neointimal hyperplasia [55].

Peroulis et al. evaluated the effect of a single intra-operative vein graft transfection of decoy binding Egr-1 in the suppression of vein graft intimal hyperplasia in hypercholesterolaemic rabbits. They treated jugular vein to carotid artery interposition grafts with the Egr-1 decoy through a nondistending pressure. The Egr-1 decoy decreased the Egr-1 gene expression by 60% as well as decreased cellular proliferation. Importantly, the Egr-1 decoy reduced intimal thickness in the grafts, whereas it was increased throughout the luminal area [58].

### 4.6. E2F

E2F is a transcription factor that has an essential role in the coordinated transactivation of cell-cycle-regulatory genes which are involved in the formation of lesions subsequent to vascular injury [59]. Morishita and his research team developed a double-stranded DNA decoy with a high affinity binding ability for E2F which can bind E2F and prevent the activation of genes that mediate cell cycle and intimal hyperplasia subsequent to vascular injury. Transfection of the E2F ODN decoy via HVJ-liposome inhibited expression of c-myc, proliferating-cell nuclear antigen (PCNA) encoding genes, and vascular smooth muscle cell proliferation in the rat carotid injury. The results from two weeks after a single transfection showed that formation of neointima was inhibited through treatment with the E2F decoy; this prevention remained up to 8 weeks after transfection in a dose-dependent manner. Therefore, it was concluded that the E2F decoy can prevent SMC proliferation and the formation vascular lesions in rat carotid injury [60].

Coronary artery bypass graft surgery with autologous vein grafting is frequently performed but progressive neointimal hyperplasia still contributes to considerable vein graft failure. Edifoligide is a decoy that binds to E2F transcription factors and inhibits its effects thus preventing neointimal hyperplasia and vein graft failure. In a phase III randomized placebo-controlled trial of 3014 patients, Alexander et al. treated vein grafts ex vivo with edifoligide. Results showed that edifoligide is no more effective versus placebo in preventing vein graft failure [61]. A total of 2865 of the 3014 patients [61] were studied for 5 years and showed that death, myocardial infarction, revascularization, and rehospitalization in the decoy group were the same as the placebo [62].

E2F-1 plays a vital role in DNA synthesis as well as progression of the cell cycle. Therefore, suppressing E2F-1 has the potential to inhibit the cell cycle [63]. Edifoligide, an E2F-1 decoy, inhibited expression of cell nuclear antigen (PCNA), the proliferation of SMCs, and can prevent atherosclerosis [64].

Ehsan et al. delivered the E2F-1 decoy to graft VSMCs of rabbits. They found that this decoy could prevent the upregulation of PCNA, medial VSMC proliferation, SMC proliferation, and reduce neointimal thickness. After 6 months, the decoy group grafts stayed without macroscopic plaque grafting compared to the control group [64].

In a randomized trial containing 41 patients with graft failure, the phosphorothioate E2F-1 decoy was delivered to the human vein grafts. It was shown that the E2F-1 decoy reduced the expression of PCNA and c-myc as well as decreased stenosis. Furthermore, there were no adverse effects in the treatment group [63].

In a randomized phase II trial containing 200 patients, Grube et al. investigated the effects of the E2F-1 decoy in coronary bypass grafting for 1 year. Decoy treatment reduced critical stenosis and neointimal volume [65].

A randomized placebo-controlled phase III trial conducted by Conte et al. evaluated the effects of edifoligide in 1404 infrainguinal revascularization patients to inhibit vein graft failure for 1 year. The improvement in secondary graft patency in the treatment group (83%) was more than the control group (78%). Therefore, the E2F-1 decoy in vein graft treatment did not provide any protection against vein graft failure [66].

Nakamura and his research team evaluated the possibility of E2F decoy treatment to inhibit neointimal formation of a balloon-injured pig coronary artery. The E2F decoy ODN was transfected after balloon inflation, followed by angioplasty. Results from the histological evaluation and intravascular ultrasound (IVUS) confirmed that the plaque area was significantly reduced by a single transfection of the E2F decoy compared to the control after one month. In contrast, the E2F decoy increased luminal and total vessel areas. The acute toxicity of the E2F decoy was tested in monkeys, and no obvious side effects were detected [67].

Kawauchi et al. evaluated the effects of the E2F decoy and antisense cyclin-dependent kinase (cdk2) kinase ODN by ex vivo single intraluminal delivery into cardiac allografts of mice and Japanese monkeys through the HVJ (an artificial viral envelope–liposome method). Results showed that E2F decoy suppressed neointimal formation in mice and prevented the expression of cell-cycle regulatory genes including PCNA, c-myc, and cyclin-dependent kinases 2 (cdk2) for up to 8 weeks, while antisense cdk2 kinase ODN had limited effects. The E2F decoy remarkably suppressed neointimal thickening and prevented cell-cycle regulatory genes in primate models whereas intimal thickening developed in the control groups. In conclusion, the E2F decoy has the potential to prevent graft arteriopathy without systemic adverse effects [68].

### 4.7. NF-κB

Nuclear factor-kB (NF-κB) is characterized as a transcription factor that upregulates adhesion molecules including VCAM-1, intercellular adhesion molecule 1 (ICAM-1), and endothelial leucocyte adhesion molecule-1 (ELAM-1) [69]. It was shown that NF-κB has an important role in vascular tissue remodeling after percutaneous coronary intervention with a stent implantation [70]. Moreover, myocardial reperfusion injury develops as a result of coordinated activation of a series of cytokine and adhesion molecule genes related to NF-κB. This results in leukocyte attachment, secretion of cytotoxic molecules, and severe damage to myocytes and endothelial cells [71].

Miyake et al. developed a chimeric decoy for simultaneous inhibition of NFkB and E2F. The results demonstrated that the chimeric decoy could inhibit VSMC proliferation and migration more than the other two decoys but had no effect on the proliferation of endothelial cells. The chimeric decoy suppressed distal and proximal anastomotic intimal hyperplasia, accelerated re-endothelialization, and inhibited macrophage accumulation. Additionally, the chimeric decoy repressed the expression of PDGF-B and PDGF receptor-b which resulted in a decreasing of smooth muscle actin (SMA)-positive cell accumulation and also reduced the expression of the VCAM-1 and monocyte chemoattractant protein-1 (MCP-1) genes [72].

In a clinical trial, Suzuki et al. studied the effect of the NF-κB decoy on the prevention of restenosis after an 8-year observation period. They transfected the NF-κB decoy at the stent site to prevent repeated restenosis. The patient (a man), received a percutaneous coronary intervention (PCI) with stents at both stenotic sites and the distal site but was not given a decoy at the proximal site. Evaluation after 4 and 8 years revealed suppressed development of neointimal formation in treated versus untreated sites. The analysis showed that decoy lesions were less frequent than the area with no decoy in year 8. Furthermore, no systemic adverse effect or late thrombosis formation was detected [70].

Kim et al. injected the NF-κB decoy ODN into the tail vein of mice with atherosclerosis (LPS/Fat-induced mice). Treatment with the NF-κB decoy ODN decreased pro-inflammatory cytokines and inflammatory markers VCAM-1 and ICAM-1. In addition, the NF-κB decoy decreased the expression of fibrosis-related proteins, TGF-β1, and fibronectin [73].

NF-κB and Sp1 are important transcription factors for the stimulation of various inflammatory genes involved in atherosclerosis. Lee et al. developed a chimeric decoy containing the NF-κB and Sp1-binding sites to suppress these transcription factors concurrently. This decoy was injected into the atherosclerotic mouse model and the results indicated that the decoy blocked the DNA-binding activity of both NF-κB and Sp1 at the transcriptional level. The serum lipid level of total cholesterol (TC) and triglyceride (TG) in the decoy-treated groups was significantly lower. Additionally, the chimeric decoy improved atherosclerotic changes, decreased inflammatory cytokines, and inhibited the expression of atherosclerotic markers [74].

Neointimal hyperplasia is one of the major disease processes in vein graft failure. A study conducted by Miyake et al. examined the effects of the NF-κB decoy on inhibition of vein graft failure. Treatment with the NF-κB decoy was intraoperatively transfected to the hypercholesterolemic rabbits. The NF-κB decoy repressed intimal hyperplasia 4 weeks after vein implantation. Furthermore, this decoy increased medial thickness which led to a reduction in intima-to-media ratio. Results also showed that treatment inhibited the macrophages recruitment and the VSMC proliferation in the neointima and increased apoptosis in VSMC [75].

Feeley et al. designed transcription factor decoys (TFD) to bind to NF-κB. The sequences of NF-κB decoys were selected from the consensus NF-κB-binding site and the promoters of ICAM-1, VCAM-1, and ELAM-1. They hypothesized that these decoys could reduce the expression of adhesion molecules, decrease reperfusion injury, decrease acute rejection, and reduce graft coronary artery disease (GCAD) in cardiac allografts in rat models. NF-κB TFD treatment reduced the expression of adhesion molecules and reperfusion injury parameters as hypothesized. Allografts treated with the NF-κB decoy had higher levels of apoptosis with survival being prolonged by decoy treatment. Moreover, allografts treated with the NF-κB decoy had decreased myointimal proliferation and intimal:medial ratios [76].

Restenosis after endovascular intervention is one of the main limitations in the treatment of cardiovascular disease [77]. Miyake et al. used a balloon catheter-based delivery system combined with chitosan-modified PLGA carriers for delivery of the NF-κB decoy into target vessels during angioplasty in order to inhibit neointimal hyperplasia in rabbits. Results showed that local application of the NF-κB decoy inhibited neointimal formation that was associated with the suppression of NF-κB binding activity. The therapeutic effects of the NF-κB decoy were carried out by macrophage recruitment inhibition via the suppression of gene expression in genes including MCP-1, VCAM-1, and CC chemokine ligand 4 as well as through the inhibition of VSMCs growth by a reduction in the expression of cyclin A and PCNA. Importantly, treatment with the NF-κB decoy resulted in the restoration of the ECM which was associated with increased expression of phosphorylated Bcl-2 in endothelial cells [77].

Tomita and his team determined the effects of the NF-κB decoy on NF-κB-mediated genes involved in the inflammatory response of endothelial cells. The NF-κB decoy was transfected into endothelial cells via a liposome-mediated method. The NF-κB decoy blocked the binding of NF-κB to its specific cis element as well as inhibited TNF-induced expression of IL-6 and ICAM-1 at the mRNA and protein level [78].

Inagaki et al. investigated the efficacy of an ultrasound-microbubble-mediated in vivo delivery for NF-κB decoy transfection in injured mouse arteries. After establishing arterial injury in murine femoral arteries by flexible wires, NF-κB decoy transfection reduced the neointima/media areas. The expression of inflammatory factors was found to be enhanced in non-treated injured arteries, whereas the NF-κB decoy inhibited the expression [33].

VSMC proliferation and migration may lead to intimal hyperplasia during vascular injury [71]. Kiomy Osako et al. designed a decoy ODN through binding the extremities of two single strands in order to reduce non-specific binding as well as degradation of the decoy ODN from the 3-terminus, called a ribbon-type decoy ODN (R-ODN). The effects of the R-ODN on inhibition of NF-κB were evaluated in human aortic VSMC. The resistance to exonucleases and stability of the R-ODN in serum were more than the nonmodified decoy ODN. Results also showed that the R-ODN could inhibit MMP-9 expression and VSMC proliferation induced by TNF-α [71].

Yoshimura and his research partners showed that in vivo transfection of NF-κB decoy into a balloon-injured rat carotid artery could inhibit neointimal formation 14 days after injury. Apoptosis and the expression of p53 were upregulated in the neointimal area in the vessels transfected with the NF-κB decoy ODN. Additionally, ICAM-1 and VCAM-1 expression, which were induced by balloon injury in the neointimal area, were decreased in vessels treated with the decoy. The migration of T-lymphocytes and macrophages into the media and neointima was also inhibited by the decoy [79].

In another study, Yamasaki et al. transfected the NF-κB decoy ODN into the balloon-injured pigs using a hydrogel balloon catheter. Treatment by decoy inhibited the proliferation of VSMC and reduced the neointimal areas 1 and 4 weeks after single transfection and was accompanied by a reduction in PCNA-positive stained cells. Furthermore, the expression of ICAM was inhibited leading to a reduction in migration and accumulation of macrophages [80].

### 4.8. Smad

TGF-β1 exerts pro-atherosclerotic effects in various vascular disorders. TGF-β1 downstream signaling involves Smads that regulate genes involved in atherosclerosis. An et al. used a Smad decoy to prevent the atherosclerosis development in the shear stress-induced ApoE-/-mice. The Smad decoy reduced the gene expression of TGF-β1 and α-SMA. The Smad decoy ODN suppressed the histological atherosclerotic changes and prevented extracellular matrix deposition in atherosclerotic mice [81].

### 4.9. TNF-Like Cytokine 1A

Tumor necrosis factor (TNF)-like cytokine 1A (TL1A), which belongs to the TNF superfamily, plays critical roles in the development of chronic inflammation [82]. TL1A receptors include decoy receptor 3 (DcR3), and death receptor 3 belongs to the TNF receptor superfamily [83].

DcR3 is an antiapoptotic soluble receptor that has pro-inflammatory functions and has a critical role in immune modulation. Chang et al. evaluated the association of circulating DcR3 levels with coronary artery disease (CAD) severity for the prediction of future major adverse cardiovascular events in 152 CAD patients. DcR3 levels were significantly higher in the high versus low and intermediate syntax score groups. Therefore, DcR3 levels were identified as an independent predictor for a high syntax score. Further results indicated that evaluated level of circulating DcR3 is associated with increased 1-year major adverse cardiovascular events in patients with multivessel CAD. Therefore, increased levels of circulating DcR3 are associated with CAD severity and could predict future major adverse cardiovascular events in patients with multivessel CAD [84].

To determine the involvement of TL1A and DcR3 in promoting atherosclerosis, Li et al. evaluated the levels of TL1A and DcR3 in the plasma of patients with CAD. Results from the multivariate analysis showed that TL1A and DcR3 levels in plasma in CAD patients were more than those of non-CAD patients. Moreover, it was shown that DcR3 and TL1A have high specificity and sensitivity for diagnosis of CAD with TL1A being positively and significantly correlated with the Syntax score in CAD patients. The circulating levels of both TL1A and DcR3 were higher in CAD patients and required more coronary artery bypass grafting than in the control subjects, thus making them potential biomarkers for diagnosing severe CAD [83].

### 4.10. Sterol Regulatory Element Binding Protein (SREBP)

SREBP controls homeostasis of lipids by regulating the expression of enzymes required for the synthesis of fatty acids, triacylglycerol, endogenous cholesterol, and phospholipids [85]. An et al. used the SREBP decoy ODN to inhibit SREBPs in high-fat diet fed hyperlipidemic mice. Results showed that the decoy could inhibit the increased expression of fatty acid synthetic pathways, including SREBP-1c, ACC1, SCD-1, FAS, and 3-Hydroxy-3-Methylglutaryl-CoA Reductase (HMGCR). Moreover, decoy treatment decreased pro-inflammatory cytokines, such as interleukin (IL)-8, IL-1β, TNF-α, and IL-6 expression [85].

## 5. Conclusions

Atherosclerosis is a systemic disease that affects arteries at different sites. Progression of atherosclerosis can lead to serious problems including heart attacks and stroke. Since traditional therapies such as statins are insufficient and sometimes are not effective, novel therapeutic strategies must be considered.

**Figure 3:**
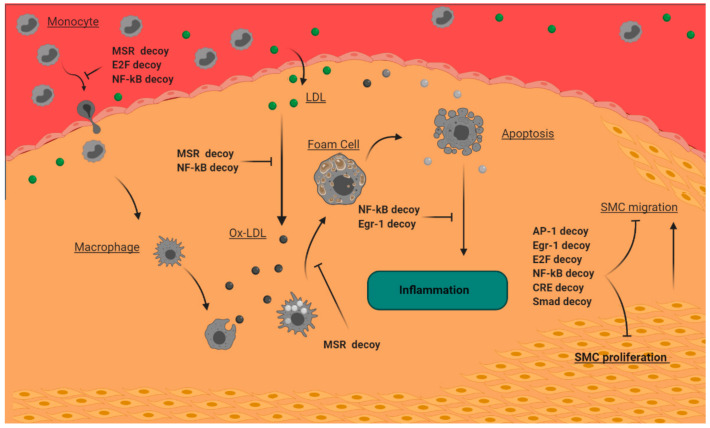
Anti-atherogenic mechanisms of decoys in the atherosclerosis progression. MSR and NF-κB decoys inhibit LDL oxidation; MSR, E2F, and NF-κB decoys suppress monocyte recruitment; the MSR decoy reduces foam cell formation; NF-κB and EGR-1 decoys reduce inflammation; AP-1, EGR-1, E2F, NF-κB, Smad, and CRE decoys inhibit SMC proliferation and migration. AP-1: activator protein-1; CRE: cyclic adenosine monophosphate response element; EGR-1: early growth response factor-1; LDL: low-density lipoprotein; MSR: macrophage scavenger receptors; NF-κB: nuclear factor-kB; SMC: smooth muscle cell.

Many studies reveal that decoy technology shows promising potentials for its great efficacy in atherosclerosis and thus must continue to be studied. In this review, we outlined two main decoy strategies, namely decoy ODNs and decoy peptides in atherosclerosis treatment studies (Table 1). Decoy ODNs have the constructs of cis elements for binding of transcription factors allowing them to selectively block key transcription factors involved in atherosclerosis pathogenesis. As therapeutic tools, synthesized decoy ODNs bind and block AP-1, CRE, EGR-1, E2F, and NF-B, and have been proven to have anti-atherosclerotic effects (Figure 3). Decoy peptides act as a molecular trap and attract the ligand of the special receptor which triggers a signaling pathway in the atherosclerosis pathogenesis resulting in the inhibition of the signaling pathway. Decoy peptides that are designed to be similar to SERCA2a and macrophage scavenger receptors were effective in the reduction of atherosclerosis symptoms (Figure 3).

Further studies are warranted to support the effectiveness of decoys and evaluate the positional side effects of them in human models. Adverse events of novel therapeutic approaches, such as decoy technology in humans, are considered to be improved through additional research. Moreover, one of the major limitations of decoy ODN technology is their stability, which significantly dampens the in vivo application. Decoy ODNs are degraded by intracellular nucleases. To date, several different types of double-stranded decoy ODNs, such as circular dumb-bell double-stranded decoy ODNs [86] and modified decoy ODNs with locked nucleic acids [87], have been developed to overcome this limitation. Another important issue related to decoy technology is the delivery strategy, which determines the in vivo efficacy and/or specificity.

## Figures and Tables

**Table 1:** Efficacy of decoy ODNs and decoy peptides in atherosclerosis.

Decoy Name	Type of CVD	Target Up/Down ↑ ↓	Concentration/Dose µM mg/kg/day	Decoy Type	Model/Cell Line	Delivery Method	Results	Ref.
ΨPLB-SE	Injury of the carotid artery (vascular proliferative disorders)	protein phosphatase 1 ↓	5 μg in 200 μl buffer injection for 15 min	Peptide	Balloon-injured rat carotid arteries	-	-Preventing the SERCA2a degradation in VSMC-reduced neointimal growth carotid artery	[44]
sMSR	Atherosclerosis	MSR ↓	2.5 mg/mL	Peptide	RAW 264 cells and peritoneal mouse macrophages	Adenovirus	-Decreased adhesion of monocyte/macrophage to the endothelial cells -Prevented the formation macrophage foam cell	[45]
sMSR	Atherosclerosis	MSR ↓	Single injection of 7.5 × 10^9^ AAVsMSR particles	Peptide	LDLR knockout mice	AAV	-Reduced atherosclerotic lesion in the aorta	[46]
sMSR	Atherosclerosis	MSR ↓	1 × 10^9^	Peptide	Hypercholesterolemic LDLR knockout mice	Recombinant adenovirus	-Reduces atherosclerotic lesion area	[47]
AP-1 decoy	- restenosis -injured carotid arteries -neointimal thickening	AP-1 ↓	15 nmol/L	ODN	-Balloon-injured rabbit carotid arteries -human aortic SMCs	HVJ liposomes	-Reduced the neointimal area. -Decreased SMCs cell number -Decreased TGF-β1 production of SMCs	[48]
AP-1 Decoy	Restenosisafter angioplasty Neointimal formation Intimal Hyperplasia	AP-1 ↓	-	ODN	Balloon-injured Rats	HVJ-liposome	-Inhibited VSMC proliferation and migration. -Abolished neointimal formation after balloon injury	[50]
AP-1 Decoy	Intimal Hyperplasia	AP-1 ↓	-	ODN	Mongrel dogs	HVJ-liposome	-Inhibited intimal hyperplasia	[51]
AP-1 decoy	Oxidative stress-induced proliferation and MMPs in rat cardiac fibroblasts	AP-1 ↓	-	ODN	Rat cardiac fibroblasts	LipofectAMINE 2000	-Inhibited XXO-induced CF proliferation and MMP gene expression	[52]
CRE decoy	Intimal hyperplasia	CRE ↓	7.814 pmol/mL	ODN	Mice	ultrasound-sonoporation	-Decreased VSMC proliferation and migration -Suppressed the intimal hyperplasia formation	[54]
EGR-1 decoy	Atherosclerosis and restenosis	EGR-1 ↓	80 µM	ODN	Hypercholesterolemic rabbits	-	-The Egr-1 decoy reduced inflammation, cell proliferation and later neointimal hyperplasia	[55]
EGR-1 decoy	Vein graft failure intimal hyperplasia	EGR-1 ↓	500 µg	ODN	Rabbits	Fugene6 transfection reagent	-Reduced VSMC proliferation and intimal hyperplasia	[56]
EGR-1 decoy	Neointimal hyperplasia	EGR-1↓	0.1 μM	ODN	Balloon-injured rat VSMCs	FuGene6	-Inhibited VSMC proliferation and neointimal hyperplasia	[57]
EGR-1 decoy	Intimal Hyperplasia	EGR-1 ↓	40 µmol/l	ODN	Hypercholesterolaemic rabbits	-	-Suppressed intimal hyperplasia	[58]
E2F decoy	-Carotid injury -Abnormal growth of vascular cells	E2F ↓	3 µM	ODN	-Rat carotid injury -Rat aortic VSMCs	HVJ liposomes	-Inhibited proliferation of SMC -Inhibited formation of vascular lesion	[60]
E2F decoy	Neointimal hyperplasia and vein graft failure	E2F ↓	0.38 mg/mL (40 µmol/L)	ODN	Human	pressure-mediated delivery system	-Edifoligide is no more effective than placebo in preventing of vein graft Failure	[61]
E2F decoy	Atherosclerosis neointimal thickness	E2F ↓ PCNA ↓	40 µmol/L	ODN	Cholesterol-fed rabbits	nondistending pressure-mediated transfection	-Reduced neointimal thickness -Inhibited plaqe formation	[64]
E2F decoy	Atherosclerosis graft failure	E2F ↓ PCNA ↓ c-myc ↓	40 µmol/L	ODN	Human	pressure-mediated DNA transfection	-Decreased stenosis	[63]
E2F decoy	Atherosclerosis	E2F ↓	40 µmol/L	ODN	Human	-	-Reduced critical stenosis and neointimal volume	[65]
Edifoligide	Atherosclerosis	E2F ↓	40 µmol/L	ODN	Human	-	-Improvement in secondary graft patency -Did not showed any protection against vein graft failure	[66]
E2F decoy	Atherosclerosis intimal hyperplasia	E2F ↓	1 mg/pig	ODN	Balloon-injured pig	hydrogel catheter	-Reduced plaque area -Increased luminal and total vessel areas	[67]
E2F decoy	Neointimal formation Cardiac Allograft Arteriopathy	E2F ↓		ODN	Mice and Japanese monkeys	HVJ	-Suppressed neointimal formation and prevented expression of cell-cycle regulatory genes -Reduced Cardiac allograft arteriopathy	[68]
chimeric decoy	Neointimal formation	NF-κB ↓ E2F↓	200 nM	ODN	Cholesterol-fed rabbits	-	-Suppressed anastomotic intimal hyperplasia -accelerated re -endothelialization -Inhibited macrophage accumulation -Repressed the expression of VCAM-1 and MCP-1 gene -Inhibited VSMC proliferation Chimeric decoy was more than two others.	[72]
NF-κB decoy		NF-B↓	600 nM					
E2F decoy		E2F↓	600 nM					
NF-κB decoy	Remodeling of vascular neointimal formation restenosis	NF-κB ↓	1 mg	ODN	Human	remedy catheter	-Suppressed the development of neointimal formation -reduced lesion	[70]
NF-κB decoy	Atherosclerosis	NF-κB ↓	0.4 mg ⁄ kg	ODN	LPS/Fat-induced mice	-	-Decreased pro-inflammatory cytokines and inflammatory markers, VCAM-1 and ICAM-1	[73]
Chimeric decoy	Atherosclerosis	NF-κB ↓ Sp1 ↓	10 µg per mouse	ODN	LPS/atherogenic diet-induced mice	-	-Decreased TG and TC -improved atherosclerotic changes	[74]
NF-κB decoy	Neointimal hyperplasia Atherosclerosis	NF-κB ↓	40 µmol/l	ODN	Hypercholesterolemic rabbits	pressure-mediated transfection	-Inhibited the development of neointimal hyperplasia -Suppressed inflammatory changes and accumulation of VSMC	[75]
NF-κB decoys (NF-ICAM, NF-VCAM, NF-ESEL)	Graft coronary artery disease (GCAD)	NF-κB ↓ ICAM ↓ VCAM ↓ ESEL ↓	160 µmol/L	ODN	Rat	pressure-mediated	-Blocked adhesion molcule expression and reperfusion injury -Prolongs allograft survival and decreases GCAD	[76]
NF-κB decoy	Restenosis Neointimal Formation neointimal hyperplasia	NF-κB ↓	-	ODN	Rabbits	chitosan-modifed PLGA NS	-Inhibited neointimal formation -Restored ECMs -Inhibited macrophage recruitment -Inhibited VSMCs growth	[77]
NF-κB decoy	Inflammation in atherosclerotic	NF-κB ↓	2 µmol/l	ODN	Mouse brain microvascular endothelial cells	cationic liposome	-Inhibited TNF-induced expression of interleukin-6 and ICAM-1 in endothelial cells	[78]
NF-κB Decoy	Neointimal Formation	NF-κB ↓	20 µg	ODN	Arterial injured mice	ultrasound-microbubble-mediated	-Reduced the neointima/media areas. The expression of inflammatory factors	[33]
R-ODN	Cardiovascular diseases	NF-κB ↓	10 nM	ODN	VSMC	lipofectamine	-Expression of MMP-9 and the proliferation of VSMC were inhibited	[71]
NF-κB decoy	Atherosclerosis /lesion formation after vascular injury/ intimal hyperplasia	NF-κB ↓	15 µM	ODN	Balloon-injured rat	HVJ-liposome	-Apoptosis was upregulated -ICAM-1 and VCAM-1 expression was decreased -The migration of T-lymphocytes and macrophages into the media and neointima was inhibited	[79]
NF-κB decoy	Intimal hyperplasia neointimal formation	NF-κB ↓	1 mg/pig	ODN	Balloon-injured pigs	hydrogel balloon catheter	-Decoy inhibited the proliferation of VSMC- Reduced the neointimal area -Decrease the expression of ICAM	[80]
Smad decoy	Atherosclerosis	TGF-β1 ↓ PAI-1 ↓ α-SMA ↓	-	ODN	Shear stress-induced ApoE-/-mice	Trans IT In vivo Gene Delivery System	-Suppressed the histological atherosclerotic changes -Prevented the extracellular matrix deposition	[81]
DcR3 (Biomarker)	Atherosclerosis	-	-	Peptide	Human	-	-Circulating levels of DcR3 in CAD patients require coronary artery bypass grafting are high	[83]
DcR3 (Biomarker)	Coronary Artery Disease Severity			Peptide			-Increased level of circulating DcR3 are associated with CAD severity and predict future MACE in patients with multivessel CAD	[84]
SREBP decoy	Atherosclerosis	SREBP-1c ↓ FAS, SCD-1 ↓ ACC1 ↓ HMGCR ↓	10 μg every two weeks for 12 weeks	ODN	High-fat diet fed hyperlipidemic mice	-	-Regulated lipid metabolism and inhibited lipogenesis -Decreased pro-inflammatory cytokines	[85]

## Data Availability

Not applicable.
